# Improving Communication of Public Health Bachelor's Degree Programs Through Visual Curriculum Mapping

**DOI:** 10.3389/fpubh.2022.770575

**Published:** 2022-05-09

**Authors:** Denise C. Nelson-Hurwitz, Michelle Tagorda, Uday Patil, Lisa Kehl

**Affiliations:** Office of Public Health Studies, Thompson School of Social Work and Public Health, University of Hawai‘i at Mānoa, Honolulu, HI, United States

**Keywords:** public health education, bachelor's of public health, undergraduate public health, high-impact educational practices, curriculum, curriculum mapping, universal design for learning, communication tools

## Abstract

Undergraduate students balance course requirements for the university, college, school, and major. Each set of requirements, including degree-specific curriculum, is intended to promote synergistic interaction of competence, skills, and knowledge, beyond serving as a collection of individual courses. Understanding of curriculum is important for program recruitment as undergraduate students are more informed when deciding between bachelor's degrees options. Among cohorted programs, this understanding is also helpful in communicating and promoting common intellectual experiences. Comprehension of curriculum is especially important for persistence when students are better able to articulate the connections between course and competencies needed to advance in coursework. To improve universal design for learning within program advising, visual curriculum maps were created as infographics to support student understanding of Bachelor of Arts in Public Health degree requirements and specific capstone course pathways. This map is printed as a small booklet and has been pilot tested among prospective students with positive feedback, then implemented in routine advising sessions. Visual maps of capstone requirements were well-received in concept, however constructive student feedback during pilot testing necessitated further revision. Student feedback also encouraged the application of culturally appropriate visuals and analogies to celebrate student diversity. Visual aids such as these may improve access to information among students through universal design, and also improve recruitment, retention efforts, and student buy-in to degree curricula.

## Introduction

Aligning competencies, courses, and skills are always challenging, but making the connection can benefit tertiary education as much as it does in primary and secondary levels (1–3). Streamlined curricula help students meet overlapping and often daunting requirements of a hierarchical academic setting, and offer a helpful tool for curricula evaluation and program accreditation (4–6). Incorporating student learning perceptions and outcomes are just a few steps toward alignment (7).

### Constructive Alignment

Curriculum itself is composed of “materials, processes and interactions comprising a course or programme of study where the goal is to provide new knowledge or skill” (1). The synergistic learning objectives yielded from the alignment of curricula are fundamental to student success. Constructive alignment, a principle of Biggs and colleagues (8), calls for a focus on the sequence of learning activities and tasks to serve student outcome goals, while the overall curriculum provides the linkages that make the sequence frictionless. In this way, the mapping of curriculum is as much a product of students as it is faculty.

Other benefits of careful alignment include preparing students to take responsibility for their journeys down academic pathways, one of the most important goals of a college instructor (9). Beyond the classroom, curriculum alignment and mapping are useful to curriculum that involves service-learning components or professional training (10). An essential objective of undergraduate public health degree programs is to be a conduit for graduates into the constantly evolving public health workforce (11, 12). In this sense, the unique role of the public health instructor closely follows the one espoused by King (13): to be “guides on the side” not “sages on the stage,” mainly because faculty cannot control the action on the many “stages” set up in the public health educational theater. And yet it behooves faculty to not simply serve as “peers in the rear,” where students can veer off course in pursuit of their own learning objectives—sometimes to the detriment of difficult but essential foundational courses.

The solution is an aligned curriculum where both learner and advisor can observe academic and professional progress at various levels continually (14, 15). Early, consistent branding and communication of an integrated learning experience are essential to student success (16, 17). It not only helps declared majors stay on track but also assists undecided students with a keen interest in health sciences to make informed decisions regarding academic pathways.

Degree programs, such as Bachelor of Arts in Public Health (BAPH), are prime candidates to benefit from organized, aligned curricula that naturally elevate and apply high-impact educational practices (HIEP) through multimodal communication via universal design of learning (UDL). Degree programs in public health often have multiple entryways and sometimes, multiple tracks or specialties, thus making singular curriculum pathways better represented by branching systems.

### Visual Communication

Visual communication methods are well-suited for the dissemination of often complex and dynamic pathways to graduation. The etymology of the word “curriculum” is apt here: it can be literally translated as “running a course” (18, 19). Public health education comprises five core disciplines (epidemiology, environmental health, services administration, health education and behavioral science, and biostatistics) that can pull students toward different specialization paths as interests develop (20, 21). Arnold and colleagues also identify various archetypes of public health students—pre-health students, previous pre-med students, future public health workers, undecided students, and idealists—which can enter and exit a BAPH program through different avenues (21).

Within the context of HIEP, visual curriculum maps can help illuminate the continual cooperation of common intellectual experiences, individual electives, service learning or internship degree requirements, and capstone sequences over the degree course (18, 22, 23). Harden, in a seminal work on building curriculum maps in medical science education (22), contends that a visual analog can help curriculum to be viewed from collective perspectives of stakeholders in higher education (e.g., student needs, content coverage, learning locations), while making transparent major learning outcomes (e.g., clinical skill, health promotion, health communication). Additionally, diversity of curricula and balances between course foci (local v. global; individual v. population) is easily ascertained.

In addition to a flowchart of required and elective classes within a department, maps can include minor milestones such as portfolio submissions, successful meetings, training certificate completion, and receipts of filed administrative forms. Multiple sets of requirements for a diverse body of students necessitate user-friendly tools such as tracking documents and dynamic websites adhering to universal design principles. Often, curriculum mapping utilizes a set of standards to ensure accessibility and usefulness.

In this paper, we present a review of efforts to produce, use, share, and evaluate visual curriculum maps to complement advising in the Bachelor of Public Health (BAPH) program at the University of Hawai'i at Mānoa.

## Pedagogical Principles

Various pedagogical principles justify the usage of visual curriculum maps while supporting and advising students. Additionally, sets of standards and competencies underlie usage and offer evidence of its relevance and reliability.

### Universal Design for Learning

The principles of UDL aim for inclusive but personalized instruction that serves the goals of students and the program alike (24, 25). This is particularly relevant as the classroom becomes more diverse and includes more non-“traditional” students from varied educational backgrounds and practicing varied learning styles (26, 27). Stylistic choices are not limited by universal design, especially when applied to visual curriculum maps. Moreover, a widely available set of simple, low-cost tools are available to help record requisite class and co-curricular information, craft visual curriculum maps, and disseminate these to students via print or digital media.

The development of the UDL framework occurred alongside the growth of undergraduate public health degree programs in the 1990's, and many of these programs are imbued with UDL's guiding principle of providing multiple means of representation, engagement, and expression (25). The inclusion of visual curriculum maps is particularly effective in aiding students to learn in alternative ways (representation) and motivating them as individual learners with unique interests, histories, and needs (engagement).

Any visual analog to the often complex instructions and myriad options during advising sessions is akin to multimodal learning in the classroom. In particular, graphical representations of complex learning pathways engage students by letting them visualize choices and forecast alternate scenarios—some of which might be of high interest but intimidating due to the hitherto unknown path. And this quasi-gamification of public health degree programs can hold great value in the long term, especially in skill-building and skill maintenance.

By removing the barriers of traditional education, public health programs are trying to foster a comprehensive understanding and retention of subject fundamentals as well as deeper expertise with the various tools of planning assessment, measurement, and evaluation. The better way of determining reception, retention, and application is to offer multiple means of activity (expression)—still a challenge to undergraduate studies at large, let alone public health programs. The end-product itself, an illustrated guide, signals to students of the varied ways to communicate information in the field. This is not unlike the infographic guides used for health promotion and patient navigation. But given the pluralistic structure and flexible materials from which this interdisciplinary subject has been constructed, there is little friction when embedding UDL principles to promote educational equity and increased motivation among students.

### CEPH Accreditation

The Council on Education for Public Health (CEPH) provides accreditation to public health programs and schools in the U.S. (28). CEPH works closely with two associations, the American Public Health Association (APHA) and the Association of Schools and Programs of Public Health (ASPPH), to ensure the quality of tertiary public health education through standardization (29, 30). Student and program competencies were determined and are regularly adjusted to reflect desired administrative, instruction, and graduation outcomes.

In this way, the advantage of visual curriculum maps can be gauged by how they help further the goal of meeting CEPH criteria and procedures, particularly in meeting requests for information (31). Core competencies of the students also become more apparent. Students, instructors, and administrators are able to see a trail of potential competencies for each degree specialization being met on a visual representation of the entire program curriculum especially when essential exercises and assignments are included. Cross-cutting competencies—those reflecting the ability of students to communicate well, practice professional behavior, embrace diversity and culture, hone leadership skills, plan programs, understand biological factors underlying common public health concepts, and embrace systems thinking—can not only be determined if they are present but also can be measured and spaced out accordingly to ensure retention well-beyond graduation.

### Goal

The underlying foundation of curriculum mapping is to communicate a program or degree curriculum as a cohesive & synergistic system/unit, including scaffolded skill development, rather than a collection of courses. This communication promotes student investment in, and understanding of, the overall curriculum and may be helpful in communicating the need for prerequisite coursework. Visual curriculum mapping, in particular, may serve to communicate individual course requirements and expectations in a more engaging and approachable manner and provides opportunities to share points of contention (hotspots) in the curriculum that require careful, thorough planning, as well as show areas of interest that inspire students to complete prerequisites and either learn or retain foundational knowledge. Omnipresent, relevant symbols and analogs, such as a train journey with stops along the route with tickets checked at specific time points, may allow for a clearer, more approachable understanding of course and curriculum connectivity and interrelationships between requirements.

The goal of this study to better communicate how overall program curricula functioned synergistically, rather than as a collection of courses, with scaffolding of both knowledge and skills. Within the broader BAPH curricula, an additional curriculum map tool was developed and applied to promote student understanding of, and buy-in to, the capstone course series at UHM. Prior to application, students expressed confusion regarding articulation of, and connections between, the three capstone courses, which are heavily scaffolded and anchored in a student-selected project topic (32).

## Learning Environment

The University of Hawai‘i system has identified itself as an indigenous-serving, Hawaiian place of learning (33). It is also classified as an Asian-American Native-American Pacific Islander–Serving Institution (AANAPISI). The University of Hawai‘i at Mānoa (UHM) is a public research university and the flagship campus for the University of Hawai‘i system. Student enrollment is 17,490 students, with undergraduate students comprising about 72.2% of enrollees (33). Campus demographics reflect a student body composed primarily of residents and high ethnic diversity (34).

The developed visual curriculum maps provide an opportunity to connect with diversity and global learning and root the BAPH degree in Hawaiian culture and include visual aids suggestive of the local environment. Specifically, within the BAPH, which has produced 319 graduates since inception (graduation rates ranging from 82 to 98% in the past 5 years), and currently serves 158 students, there is high ethnic diversity and a high proportion of first-generation college students. Navigating a university system is often challenging among students who are under-represented in higher education (35), and diverse students also commonly struggle with belonging (36–38). Visual curriculum maps provide an opportunity to explain often complex program requirements in an alternate, and often more familiar format for students.

### Development Process and Methods

The overall bachelor's curriculum was developed and launched in January 2014, and had since been communicated to students using a university standard, table-formatted program sheet, listing degree requirements, including both required and elective courses. For reasons described in the Pedagogical Principles, visual curriculum maps were developed to improve communication of both overall degree and course requirements, as well as to promote student buy-in to the curriculum, based on discussions among faculty regarding curriculum milestones to communicate with students.

Visual curriculum analogies and metaphors were conceptualized during discussions with students during routine academic advising and in courses. Application of these analogies to visually represent course requirements was occasionally pilot tested using free-hand drawings on a classroom whiteboard and anecdotally received positive feedback from students. Feedback from students was collected through informal focus groups over two separate semesters. Students expressed an improved understanding of the course, and in some cases, course series curriculum at the conclusion of the in-class discussions, but also described the analogies and discussions as both memorable and supportive in formal evaluations, specifically programmatic exit surveys and in focus groups conducted just prior to graduation.

Visual curriculum map products, (i.e., print materials), were initially developed using slideshow development software, in a similar format as an academic poster. However, the need for a wider range of accessible graphics, and graphic design flexibility required a transition to a web-based infographic design software system. Visual curriculum analogies were translated from conceptual, free-hand whiteboard drawings to formalized graphic presentations using the infographic design software, then distributed to faculty and student support staff for feedback. Based on preliminary feedback from these stakeholders, primarily centered on formatting and clarity, revisions were made. The visual curriculum maps were then printed and pilot tested among individual and small groups of target audience students. The students shared positive feedback, primarily noting appreciation for clarity of tangible steps, courses, and assignments. Minor edits were suggested, primarily highlighting opportunities for clearer connections and misalignments in formatting. After edits were made, visual curriculum maps were printed and widely distributed at strategic time points in academic advising and in course curriculum, (e.g., upon program entry, prior to beginning the capstone course series, and at the beginning of relevant coursework).

## Development and Assessment of Tools

**Figure 1:**
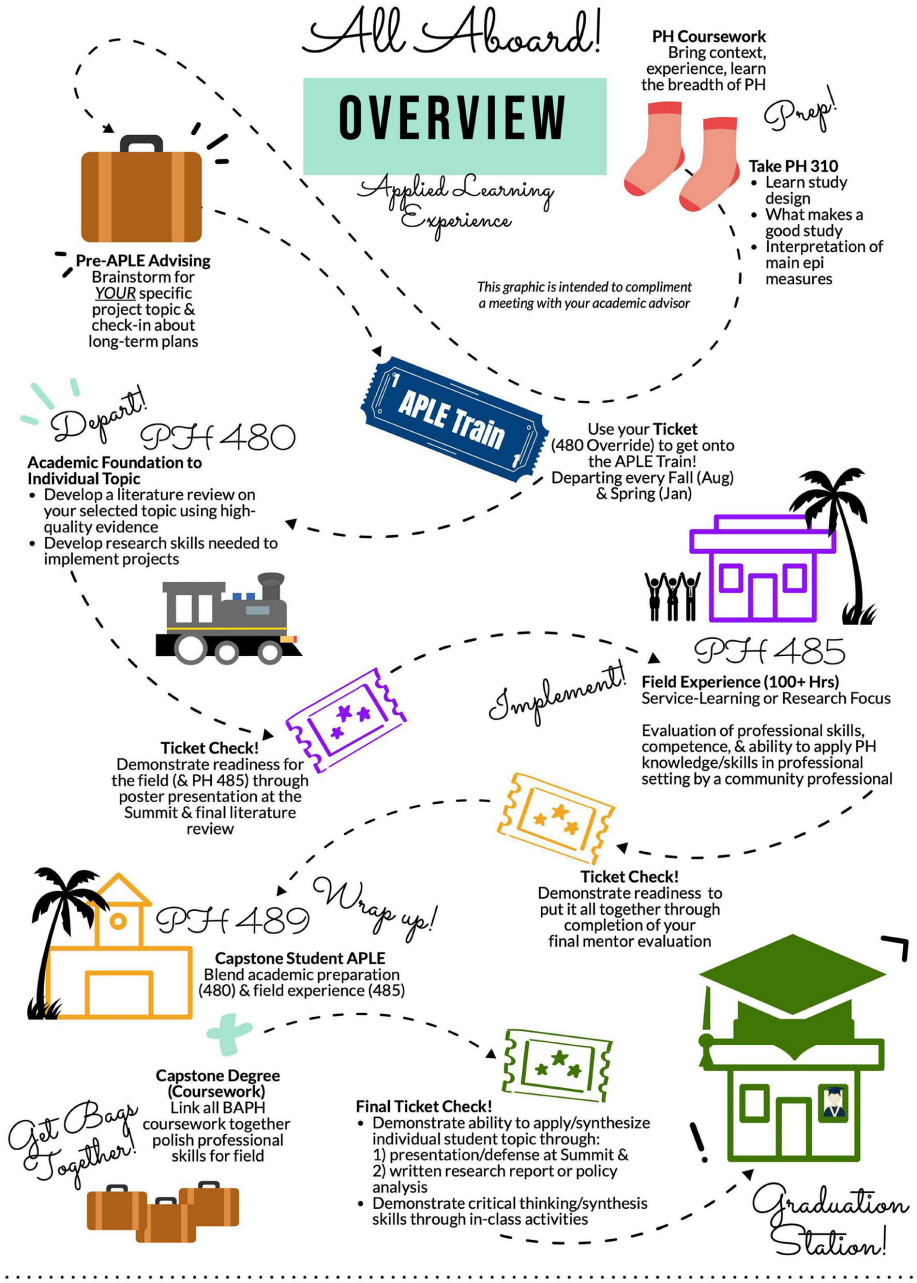
Visual curriculum map for three-course capstone experience applying train analogy.

To date, two visual curriculum maps have been developed. The visual curriculum map for the overall Bachelor degree program is available through Figshare (Supplementary Figure 1). The expanded curriculum map for the three-course capstone experience, which includes writing-intensive coursework and a service-learning or internship component (32, 39, 40), is presented in Figure 1. Since the launch of these visual curriculum maps, anecdotal evidence collected through informal focus groups and formal evaluations, specifically programmatic exit surveys and in focus groups conducted just prior to graduation, suggests students have found them to be useful tools and are able to better articulate their understanding of the curriculum during academic advising sessions. Some students found the condensed visual representations overwhelming, suggesting the need to spread maps out over multiple pages, add more white space, and provide opportunities for students to zoom in and out, (implying the need for digitization of currently printed communication tools). Student feedback also encouraged the application of more culturally appropriate analogies as an approach to celebrating student diversity. Faculty throughout the BAPH program reported students entered, and progressed, through their courses with fewer questions and less confusion regarding course articulation and scaffolding.

## Discussion

The development of visual curriculum maps is a low-cost intervention to promote a better understanding of degree and course requirements among students in an approachable and engaging manner applying principles to promote universal design for learning. Improved understanding may lead to increased self-efficacy and buy-in from students to the curriculum and degree process, with implications for both persistence and recruitment.

Visual curriculum maps also have the potential to play a critical role in the application of multiple high-impact educational practices. In working with learning communities or cohorted programs, consistent, clear, and unified requirements and processes, such as those communicated through visual curriculum maps, may support the development of common intellectual experiences among peers, even while students may be working through individual project topics. Clear and consistent communication tools may further promote opportunities for students to support each other as peers, though specific topics selected for projects or assignments may vary.

Application of visual curriculum maps has great potential in writing-intensive courses, to improve communication of how scaffolded, supportive assignments, such as outlines or annotated bibliographies, may contribute to the construction of more substantial written assignments.

### Future Directions

There is further opportunity for students to better understand connections between course assignments to both the writing process and to the development of their own writing skills. Three additional visual curriculum maps are currently in development. This includes one for an undergraduate introduction to global health course with the intent to explain the scaffolding of assignments to develop a Model United Nations policy paper and subsequent Model United Nations requirements associated with the course.

A second curriculum map is in development to replace the existing map communicating the overall Bachelor degree program curriculum with a more culturally appropriate, and comprehensive, visual representation applying an analogy of the degree program with the Native Hawaiian *ahupua‘a* system of land division and water movement (41, 42). This more comprehensive curriculum map would incorporate gamification strategies and require students to collect required coursework or other milestones as represented by foods or natural resources as they journey *mauka* (from the mountain) to *makai* (to the ocean) on a quest to embark on a voyage toward their future launching at the time of graduation. This comprehensive map will additionally include sub-maps, pull-outs, and opportunities for students to zoom in on individual courses.

## Conclusion

Visual curriculum mapping as applied here, evidenced by student and faculty feedback, and in medical science (18, 22, 23) has been an effective tool in communicating degree requirements. Through the application of visual curriculum maps, students in this study report an improved understanding of the overall capstone process, including stronger comprehension of the role played by preparatory coursework and skill development required prior to service learning or internship experience. Improved communication of both required preparation and process allows students to develop more intentionality and self-authorship in their learning process and promotes the value of student-centered learning throughout the capstone experience and overall bachelor's curriculum.

## Data Availability Statement

The original contributions presented in the study are included in the article/Supplementary Material, further inquiries can be directed to the corresponding author.

## Author Contributions

DN-H and MT contributed conception and design of the project. DN-H designed all tools. DN-H, MT, and LK implemented tools and collected evaluation feedback. DN-H and UP wrote the first draft of the manuscript. All authors wrote sections of the manuscript, contributed to manuscript revision, read, and approved the submitted version.

## Conflict of Interest

The authors declare that the research was conducted in the absence of any commercial or financial relationships that could be construed as a potential conflict of interest.

## Publisher's Note

All claims expressed in this article are solely those of the authors and do not necessarily represent those of their affiliated organizations, or those of the publisher, the editors and the reviewers. Any product that may be evaluated in this article, or claim that may be made by its manufacturer, is not guaranteed or endorsed by the publisher.
