# Supplementary material for: Improving Communication of Public Health Bachelor's Degree Programs Through Visual Curriculum Mapping
Source: Front Public Health. 2022 May 9;10:770575. doi: 10.3389/fpubh.2022.770575 (PMC9125020; doi:10.3389/fpubh.2022.770575)
Supplement: Supplementary file 1 [file Image_1.PDF]

Bachelor of Arts in Public Health

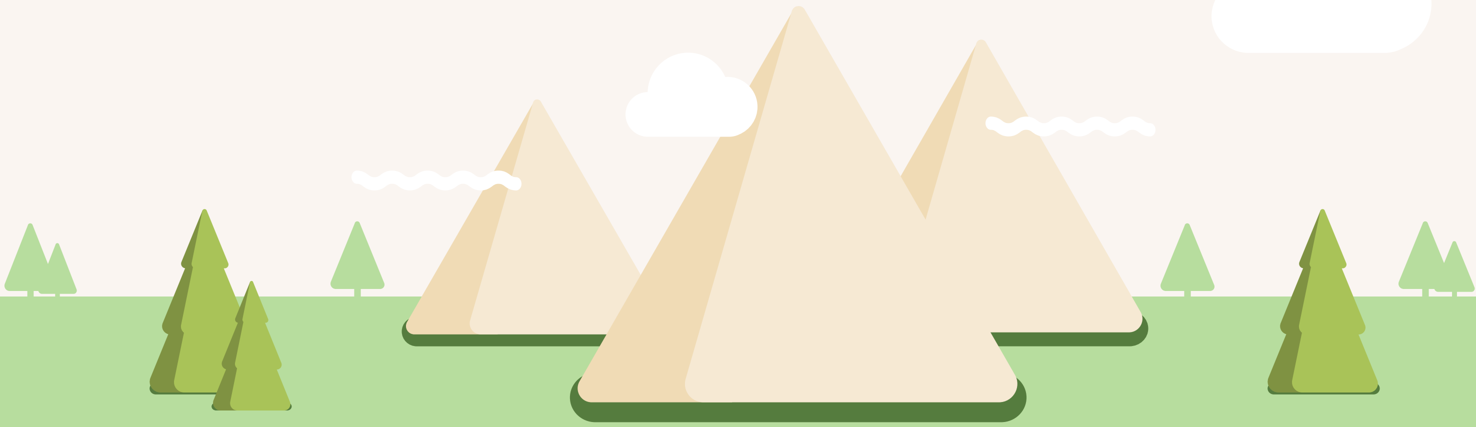

CURRICULUM OVERVIEW

Intended to Compliment Meeting with an Academic Advisor

Overall Degree Design

PH BREADTH

Coursework

Provides students with foundational knowledge and skills needed for professional practice

Prepares students for graduate degree programs or employment

PH DEPTH

Applied Learning Experience

Allows students the opportunity to personalize their degree program based on their individual interests

Provides field experience in either a service learning or research based context (student's choice)

INTRODUCTORY CORE

Courses Intended To:
Create a solid foundation for public health learning, practice, and application
Provide 3 opportunities for student exposure/repetition of public health skills & concepts
Allow students to apply skills and concepts on both a macro and micro level

PH 201 → **PH 202** → **PH 203**

INTRODUCTORY CORE

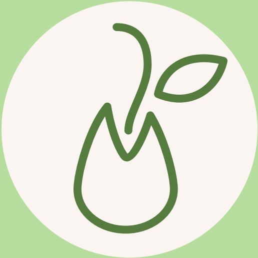

PH 201

Introduction to Public Health

Introduces students to foundational skills and concepts in public health, including the 5 core areas & cross-cutting disciplines

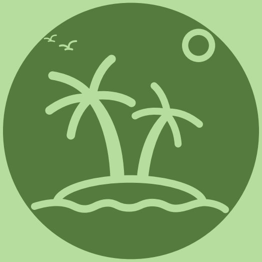

PH 202

Public Health Issues in Hawai'i

Zoom in... students apply foundational public health skills & concepts to local public health issues in Hawai'i, with an emphasis on Native Hawaiian health & oral communication skills
Prereq: PH 201

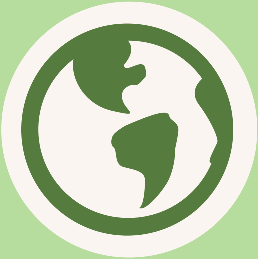

PH 203

Introduction to Global Health

Zoom out... students apply foundational public health skills & concepts to global issues & United Nations governance modeling with an emphasis on written communication skills
Prereq: PH 201

QUANTITATIVE SKILLS

Courses Intended To:
Promote development of quantitative skills most directly relevant to public health application
Prepare students for research skills necessary for application to academic project development & field experience

PH 210 → **PH 310** → **APLE**

QUANTITATIVE SKILLS

PH 210
Quantitative Reasoning for Public Health

Foundational quantitative skills intended to best prepare students for success in public health, specifically as it relates to application in epidemiology

PH 310
Introduction to Epidemiology

Students learn calculation and interpretation of primary epidemiological measures and the essentials of study design needed for critical analysis of study methodology
Prereq: PH 201, & PH 210 or Math 140+

PH 410 - OPTIONAL ELECTIVE
Advanced Epidemiology (WI)

Seminar-based discussion of epidemiological skills and concepts as used in research and practice
Prereq: PH 201 & PH 310

SOCIAL & BEHAVIORAL SKILLS

Courses Intended To:
Provide theoretical, and applied, context to public health programs and interventions intended to promote changes in individual or group behavior

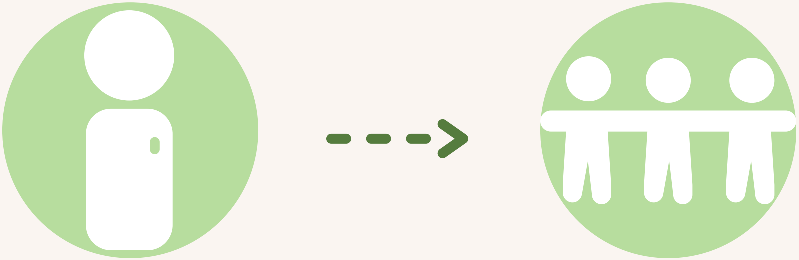

PSY 100 **PH 420**

SOCIAL & BEHAVIORAL SKILLS

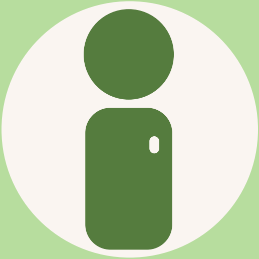

PSY 100

Survey of Psychology

Students are introduced to basic psychological theories and prepare for public health-related application of behavior change theories

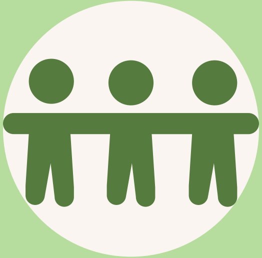

PH 420

Social Behavioral Health I: Health Promotion for Individuals & Groups

Provides a foundation in health education and social behavioral health sciences, including application of behavioral theories to program design, implementation, & evaluation
Prereq: PH 201 & PSY 100

BIOLOGICAL SKILLS

Courses Intended To Provide Biological Context to:
Onset, and progression, of infectious and non-communicable diseases
Environmental health challenges, including climate change, & assessment of natural resources

PH 341

Public Health Biology & Pathophysiology

Provides a biological foundation to understanding disease development & progression, and provides biological context to epidemiological surveillance & public health interventions
Prereq: PH 201 & one of the following: BIOL 101 or BIOL 171 or BIOL 172 or PHYL 103 or PHYL 141 or FSHN 185

PH 340 - OPTIONAL ELECTIVE

Public Health & the Environment

Understand impacts of the natural, and built, environment on human health, and apply this understanding to interventions to manage environmental risk

PH 445 - OPTIONAL ELECTIVE

Introduction to Environmental Microbiology

Students learn context to the role of microorganisms in the natural environment, and evaluation of the effect of these microbes on human activities and health
Pre: MICR 130 or MICR 351 or BIOL 171

PUBLIC HEALTH ELECTIVE COURSES

12 Credit Requirement

Opportunity for students to diversify their public health experience and personalize their public health skill set based on both personal and professional interests

Students can take PH Elective courses in the department and/or outside the PH department. For a complete list of approved public health electives offered outside of the public health department, please see the list of "Other Public Health Electives" on our website (Academics -> Degrees -> Bachelor of Arts)

APPLIED LEARNING EXPERIENCE

Student-driven capstone experience to the BA Public Health degree based in a 3-course series

PH 480

PH 485

PH 489

A mandatory advising session is required to enroll in PH 480.
See your Academic Advisor for more information.

AT GRADUATION

STUDENTS WILL...

- Be Prepared for
  - Entry-Level Employment
  - Graduate School
- Demonstrate public health skills and knowledge
- Have both a breadth of knowledge & practical field experience

BACHELOR'S
MASTER'S
DOCTORAL
CEPH
ACCREDITED

Office of Public Health Studies
Myron B. Thompson School of Social Work
University of Hawai'i at Mānoa

Updated October 2019
